# The journal *Investigación y Educación en Enfermería* receives the Family and Community Nursing Chair Award for “Recognition of the Scientific Development of Family and Community Nursing” from Universidad de Alicante in Spain

**DOI:** 10.17533/udea.iee.v43n1e17

**Published:** 2025-04-29

**Authors:** José Ramón Martínez-Riera, María de los Ángeles Rodríguez-Gazquez

**Affiliations:** 1 Nurse, Ph.D. Director Healthy University Secretariat, Universidad de Alicante, Spain. Email: catedraefyc@gmail.com https://orcid.org/0000-0002-4926-6622 Universidad de Alicante Universidad de Alicante Spain catedraefyc@gmail.com; 2 Nurse, Ph.D. Editor in Chief of Investigación y Educación en Enfermería. Email: revistaiee@udea.edu.co https://orcid.org/0000-0002-4329-4286 Universidad de Antioquia Colombia revistaiee@udea.edu.co

Last 13 December 2024 the awards ceremony for the Chair of Family and Community Nursing was held by Universidad de Alicante (Spain). This organization’s director, Doctor José Ramón Martínez-Riera, addressed the following words to the public when recognizing the journal *Investigación y Educación en Enfermería* for its support to the scientific development of family and community nursing: 

“The stimulus, support, and implication of organizations or institutions and those who represent them, are essential for the development and consolidation of Nursing through the dissemination of its research, projects, or experiences. As a scientific discipline, Nursing requires the contribution and dissemination and projection of its findings to both the scientific community and society at large. Beyond the researchers and the academic institutions or the scientific societies to which they belong, there are the scientific publications that, with the effort, perseverance, will, and attitude of those who direct and promote them can survive within a complex competitive environment, not always fair, of market and business, placing the criteria of quality, excellence, and contribution to Nursing above the business it represents for others. This is the case of the journal *Investigación y Educación en Enfermería* from the Faculty of Nursing at Universidad de Antioquia in Medellín, Colombia and in a very special way of the person who has been and continues to be the architect of its development. Dr. María de los Ángeles Rodríguez-Gázquez has been one of the pioneers in constituting a publication of these characteristics as a reference for nursing science and for this reason, the Chair of Family and Community Nursing wishes to recognize the journal *Investigación y Educación en Enfermería*.”

After these words, a video was presented with the communication by the Journal’s Editor, which stated:

“I am María de los Ángeles Rodríguez, for 15 years I have had the honor of being the editor of the journal Investigación y Educación en Enfermería of the Faculty of Nursing at Universidad de Antioquia in Colombia, which has been one of the biggest academic challenges in my life that implies the effort of maintaining and improving a journal’s high quality, which when I received it was already known throughout Iberian America.

Together with the Editorial Committee, we have worked strenuously to improve the dissemination of the knowledge contained in our pages to be accessible globally. Currently, eight of every ten articles are from outside Colombia, with significant contribution from Latin America and a large increase of manuscripts received from countries in Europe, Asia, and Oceania. In all the articles, it is evident that the methods nurses use to provide care to people around the world are similar. 

We have also improved our journal’s visibility in high-impact bibliographic databases, and we are indexed in Medline, in PubMed Central, as well as in the WoS JCR citation databases in the 3^rd^ quartile and Scopus SCImago Journal Rank in the 2^nd^ quartile, which is the result of our commitment to editorial work of excellence, besides the new challenges of digital translation and alignment with open science policies so that nursing knowledge is shared with everyone.

On behalf of the Editorial Committee members, the authors, reviewers, readers, and as editor of the IEE journal, I thank the Chair of Family and Community Nursing at Universidad de Alicante for the honor of receiving the *Recognition to the scientific development of family and community nursing* **.**

Thank you very much”

